# A Novel Approach for Weed Type Classification Based on Shape Descriptors and a Fuzzy Decision-Making Method

**DOI:** 10.3390/s140815304

**Published:** 2014-08-19

**Authors:** Pedro Javier Herrera, José. Dorado, Ángela. Ribeiro

**Affiliations:** 1 Centre for Automation and Robotics, CSIC-UPM, 28500 Madrid, Spain; 2 Institute of Agricultural Sciences, CSIC, 28006 Madrid, Spain

**Keywords:** precision agriculture, weed species discrimination, fuzzy decision making strategy, colour segmentation, Hu invariant moments, geometric shape descriptors

## Abstract

An important objective in weed management is the discrimination between grasses (monocots) and broad-leaved weeds (dicots), because these two weed groups can be appropriately controlled by specific herbicides. In fact, efficiency is higher if selective treatment is performed for each type of infestation instead of using a broadcast herbicide on the whole surface. This work proposes a strategy where weeds are characterised by a set of shape descriptors (the seven Hu moments and six geometric shape descriptors). Weeds appear in outdoor field images which display real situations obtained from a RGB camera. Thus, images present a mixture of both weed species under varying conditions of lighting. In the presented approach, four decision-making methods were adapted to use the best shape descriptors as attributes and a choice was taken. This proposal establishes a novel methodology with a high success rate in weed species discrimination.

## 1. Introduction

The Precision Agriculture (PA) concept advocates for the adjustment of resources and agronomic practices to the requirements of the soil and crop, seeking greater sustainability and efficiency. Among the practices associated with a PA schema, site-specific weed management is effective in decreasing herbicide costs, optimising weed control and preventing unnecessary environmental contamination [1–4]. Several authors have shown that the distribution of the most harmful weeds for a particular crop is not uniform, generally affecting less than 40% of the crop [5,6]. However, weeds are usually managed uniformly across the whole field, with consequent damage to the environment and waste of money. The variable spatial distribution of weeds must, therefore, be considered in weed-management strategies: by using target chemical applications, agriculture and the environment can be more sustainable [7].

To carry out suitable site-specific weed management, it is essential to have accurate information on within-field variation of weeds, *i.e.*: (*i*) where the weeds are located; (*ii*) the weed seedling density; and (*iii*) the type of infestation present. This information can be obtained by different methods, including cameras located on aerial platforms or ground platforms.

Nowadays the increasing technology of satellite and aerial images is demanding solutions for different image-based applications where it is often useful the classification of the different textures underlying the images. In this context, textures can be useful for distinguishing weeds from crop. So far, the identification of agricultural textures in aerial images and data coming from satellites has been realized by means of strategies that require a costly field sampling [8,9]. Besides, information collection depends heavily on the weather (no clouds or fog) and, although remote sensing in agriculture has experienced a resurgence in recent years due to the use of hyperspectral and multispectral cameras [10], it is expensive and produces low-resolution images. In general, satellite images are better suited for large, contiguous areas, while aerial images are better for smaller or non-contiguous areas. Satellite images may be too expensive for small survey areas, but covers a relatively large geographic area compared to aerial images. In contrast, Unmanned Aerial Vehicles (UAVs), commonly known as drones allow a higher resolution, but many countries have passed legislation that limits the use of UAVs. In addition, the less dangerous drones also have a less energy autonomy, flying on average for less than half an hour, which limits the size of area covered by the inspection. On the other hand, the images acquired from ground platforms also allow a high (centimetre) resolution, but the information contained in each image only covers a small crop area. Nevertheless, treatments are realised at ground level and this largely justifies the convenience of making detection from ground platforms.

The development of methods for weed detection from images is a very important open field for PA [11–15] and a challenge that has no simple solution owing to the great diversity of crops and weeds, changes in ambient lighting, differences in the texture of the terrain (fundamentally due to humidity), different growth states of crop and weed infestation, and great similarity of the crop and weeds [16,17]. Many techniques for weed detection have in common the segmentation of vegetation against background. They mostly take into account the fact that all pixels belonging to vegetation (crop or weed) have a strong green component. Based on this, the vegetation is separated from background (soil) independently of the type of crop treated. This characteristic can be used directly through the RGB colour model or creating colour indices that represent the “greenness” of a given pixel [12,18]. These indices are designed to cope with the variability of lighting conditions, and all these strategies oriented toward green detection need to fix a threshold for final segmentation.

These difficulties make the discrimination between the crop, weed and soil a complex task, and the difficulty increases when the objective is to discriminate between weeds species or to apply herbicide in real time as the position of the infestation is detected [1,19–23].

Precision treatment of weeds leads to the application of well-adjusted doses of herbicides directly to the target at the seedling stage. The effectiveness in controlling weeds and crop yield can be significantly improved if, in addition to precisely locating herbicides, the herbicides are applied early in the weed growth cycle (*i.e.*, the seedling stage) [24–26]. Nevertheless, the lack of commercial availability of precision application equipment continues to be an issue preventing the technology from reaching its full potential due to limitations in robustness in a wide variety of field conditions, including fluctuating weather and changing plant canopy and structure [27]. In addition, targeted recognition and application technology for precision weed control must be easily incorporated into current systems or used as stand-alone implements. While several research studies and a few commercial grade systems are being developed for targeted applications, little is known about the precise rates of herbicides and other treatments needed to control very small weed seedlings.

**Figure 1.**
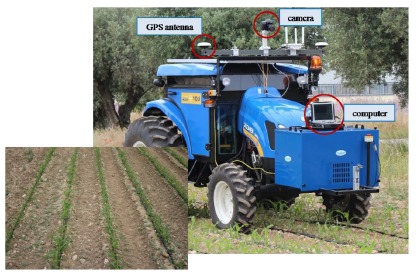
A RGB camera on board an autonomous tractor acquiring geo-referenced images in real conditions (lighting and tractor speed) in a maize crop.

**Figure 2.**
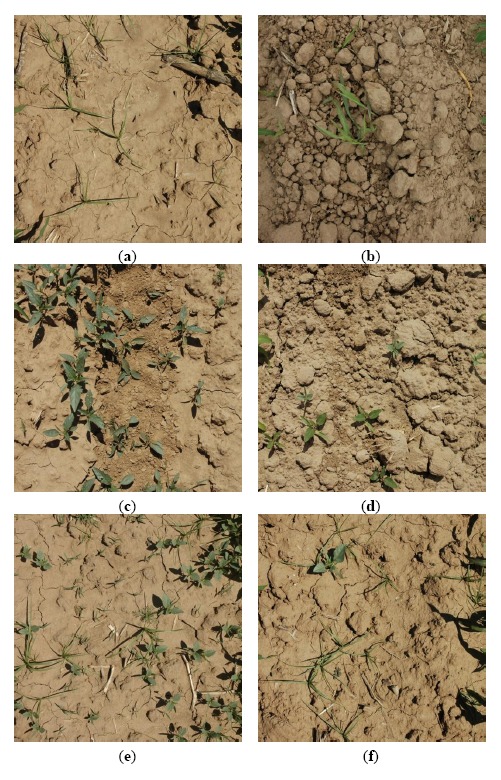
Images (**a**,**b**) show monocots (long and slender leaf), whereas images (**c**,**d**) present dicots (broadleaf and short). Images (**e**,**f**) display a mixture of both weed species.

The use of high-tech machinery to target individual weeds in real time would provide valuable tools for weed control in the field at any time [28]. For this reason, it is essential to analyse the viability of the real time performance of the proposed image processing. The final aim of this work is to integrate a RGB camera as part of the sensors systems on board an autonomous tractor in order to reach the weed classification in real time. Figure 1 illustrates the location on a tractor of the equipment used in the initial experiments conducted to analyse the performance of our approach. In this case, the camera (an EOS 7D, Canon, Tokyo, Japan) is connected to a computer by a USB port; the computer acquires images at a rate of 6 frames per second; the images are geo-referenced during the acquisition process. The type and aspect of the images obtained are shown on the bottom of Figure 1. The modification of the image perspective is performed following the process described in [29]. Furthermore, crop rows that appear in the image are eliminated by a process based on the method proposed in [30] to isolate the vegetation cover areas between the crop rows. After applying the previous process, the images obtained are similar to those shown in Figure 2. Therefore, the main objective of this work is to design, develop and assess a strategy of discrimination between weed species, able to work in real time with images as those shown in Figure 2. Finally, the developed method will be integrated on the computer on board the tractor with the aim to take effective treatment decisions.

Herbicide application efficiency would be higher if selective treatment is performed for each type of weed instead of using a single broadcast herbicide [31,32]. Most studies during the last twenty years have addressed the classification of only two classes of plants, either crop or weed, or distinguished between weeds species, broadleaf and grasses [31,33,34], e.g., based on their heights [33,34]. Broad-leaved weeds and grasses are distinguished because the selectivity of some herbicides is based on differences between monocots and dicots. Therefore, the characterisation of the spatial distribution of both groups is essential to the development of an autonomous system for treatments that can adjust the type of herbicide and the dose to the dominant infestation. However, precisely classifying a plant species that may be combined with other different species is challenging from the point of view of image processing.

The success with which these aspects can be adapted to classification depends on the type of crop and weeds, and how and when images are gathered. In other words, early weed detection in row crops is an objective that can be planned according to criteria oriented to two different levels with an increasing requirement: (1) estimation of the presence or absence of weeds according to its location in the bare soil or in the crop rows and (2) differentiation between weeds species (e.g., monocots *vs.* dicots) according to discriminant parameters (e.g., spectral characteristics, size, and shape). Previous works have tackled this problem. For example, the performance of three neural-network models for classifying images from six classes of plants is presented in [35]. The images were taken with controlled lighting and the different species always appeared isolated. A promising method based on using ultraviolet (UV) induced fluorescence is proposed in [36]. In latter case, experiments were conducted with plants grown in greenhouse. A complex method that combines well-known image processing techniques, clustering and genetic algorithms to extract leaf shapes and discriminate plant species is presented in [37]. The previous method is hardly suitable for real-time processing.

Furthermore, shape descriptors are used in many computer vision tasks [38]. In general, descriptors describe a given shape so that descriptors for different shapes should be different enough that the shapes can be discriminated. Regions can be either described by contour-based properties or by region-based properties [39]. Invariance with respect to translation, rotation and scaling is demanded in object recognition applications, whose aim is to identify an object independently of its position, orientation and size in the scene [40,41]. Hu defined in 1962 [42] a set of seven invariant moments for two-dimensional objects derived from the second and third central moments that have proved to be useful in many pattern recognition tasks [40,41]. In addition, geometric shape descriptors assess the geometric shape of the contours of the regions, e.g., the perimeter, the diameter, the eccentricity, *etc.* [38,39]. For instance, as the spatial distributions of weeds are unique, with monocot infestations being patchier than dicot infestations [6] and monocots differing structurally from dicots (as can be seen in Figure 2), a strategy based on the use of shape descriptors may be suitable for the recognition of plant shape.

In [43], is proposed new shape features based on a skeleton operation for discriminating weed/crop but is necessary to adapt the method to the varying field conditions, and improve the detection of late growth stages and overlaps in the images. In [44] plant seedling recognition is addressed by means of two approaches of shape feature generation based on plant silhouettes. The performance assessment is based on the classification accuracy of four different classifiers (KNN, Naive-Bayes, Linear SVM, Nonlinear SVM), but a description about how shape descriptors are used for each classifier is not included. Moreover, is necessary to adapt the method to the varying conditions existing in real field situations, since the images were taken with controlled lighting and the plants appear isolated. A preliminary study in [45] shows the Hu invariant moments obtained from regions that belong to the two weed species under study. The data from monocot weeds show that Hu moments tend to have negative values in the fifth, sixth and seventh moment, while the moments that give a positive value are close to 1 or 2. In dicot weeds, the values in the seven moments are close to zero and never reach 1. The fifth, sixth and seventh moments may be negative, but these values are very close to zero. Therefore, these results suggest that the Hu moments could be a valid base to appropriately characterise different weed species.

Summarizing, this work presents a novel method of discrimination between monocot and dicot weeds in images taken in real field situations and, consequently, which display a mixture of both types. The proposed method assumes that each region belonging to weeds can be characterised by a set of thirteen attributes based on the seven invariant Hu moments and six geometric shape descriptors (*perimeter, diameter, minor axis length, major axis length, eccentricity* and *area*). Based on these attributes, each region can be classified as monocots or dicots using the appropriate decision method. For the final validation, several decision-making methods have been analysed. In particular the Choquet fuzzy integral (CFI), the Sugeno fuzzy integral (SFI), the Dempster-Shafer theory (DES) and the fuzzy multicriteria decision making (FMCDM). Each one of them has been reported to give excellent results as a classifier combiner [46,47]. Moreover, based on the conclusions reported in [48–50], each strategy appears as a suitable method for the combination of attributes. In fact, with a little adjusting they can be used for combining attributes in this proposal, in outdoor images under similar characteristics (lighting condition, shadows, occlusions, *etc.*) [46–50]. Furthermore, support Vector Machines (SVM) is used in this work for comparative purposes because it has been successfully applied for crop/weed discrimination with features related to colour, texture and shape [44,51–53], although in a different context because plants appeared isolated in the images [44,52,53]. A discussion about the SVMs performance is presented in [51].

The organisation of this paper is as follows: Section 2 describes the proposed approach, including a brief overview of the decision-making strategies studied and how they are adjusted to be applied to combining attributes; moreover, the section includes a brief description of the images tested to assess the proposal performance, as well as the characteristics of the sensor and the setting of the camera used in the acquisition process. Section 3 analyses the performance of the method proposed. Section 4 presents the conclusions and future work.

## 2. Methods and Material

**Figure 3.**
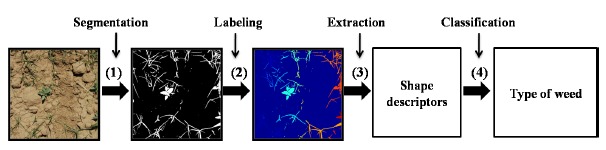
The proposed approach consists of four stages.

The proposed approach consists of the following four stages: (1) segmentation of vegetation cover and definition of regions; (2) labelling of disconnected regions; (3) extraction of the seven Hu invariant moments and six geometric shape descriptors for each region; and (4) classification of both monocot and dicot regions by means of a decision-making method; in this paper, several decision-making methods are analysed. Figure 3 shows the described process.

### 2.1. Segmentation of Vegetation Cover and Definition of Regions

**Figure 4.**
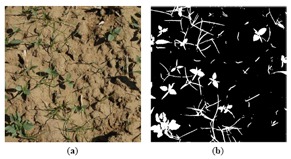
(**a**) Mixed monocots and dicots; (**b**) Segmentation of the vegetation cover of image (a).

The segmentation of the vegetation cover is a two-steps process. First, a linear combination (Equation (1)) to each pixel of the original image (*I*) is applied to create a colour index:

(1) GI=r⋅I(R)+g⋅I(G)+b⋅I(B)

where *r* = −0.884, *g* = 1.262, *b* = −0.311 [23]. These coefficients were found using a genetic algorithm optimization, and proved to perform better than Excess Green coefficients (*r* = −1, *g* = 2, *b* = −1), on similar images. Then, the original RGB image is transformed into a one-dimensional grayscale image (*GI*). The values with which these coefficients are set is the key to obtain the most appropriate segmentation of vegetation/soil, and is largely discussed in [12]. The resulting *GI* image is binarised using a threshold, which is set to 10 in this case to cope with the variability of daylight conditions [12,30]. Figure 4b shows the binarised image from Figure 4a.

Next, to enhance the regions, an opening morphologic operation is conducted to avoid overlap among regions belonging to different plants and to remove the pixels due to noise with minimum alteration of the pixels from vegetation cover. To obtain the isolated regions, the opening operation is accomplished with a structural element that symmetrically operates in all spatial directions, *i.e.*, the classical 5 × 5 matrix of ones. The election of this structural element is based on the resolution of the images and the size and characteristics of the weeds aim of the study. Moreover, the selection of this window size is consistent with the real-time requirements.

### 2.2. Labelling of Disconnected Regions and Extraction of the Hu Invariant Moments

**Figure 5.**
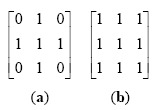
Two types of connectivity: (**a**) 4-connectivity; (**b**) 8-connectivity.

Labelling is an operation to identify distinct self-contained objects of a binary image. It can be defined two types of connectivity, 4-connectivity and 8-connectivity as it is shown in Figure 5. The connectivity determines which pixels to include in the object (region in this case).

In the second stage, the regions are labelled following the algorithm described in [54], which finds the connected components in a binary image as follows:

(1) Run-length encode the input image.
(2) Scan the runs, assigning preliminary labels and recording label equivalences in a local equivalence table.
(3) Resolve the equivalence classes.
(4) Relabel the runs based on the resolved equivalence classes.

In this method, all pixels in the same region are assigned the same level using 8-connected labelling. The connected components are searched in top-to-bottom scan order, *i.e.*, all pixels in the first connected component are labelled as 1, those in the second as 2 and so on.

Once all regions have been labelled, the seven Hu invariant moments and the following six geometric shape descriptors are computed for each region as follows:

(1) *Perimeter*: the distance around the boundary of the region (the pixels on the inside of the object's boundary).
(2) *Diameter*: specifies the diameter of a circle that circumscribes the region.
(3) *Minor axis length*: is the length (in pixels) of the minor axis of the ellipse that has the same normalized second central moments as the region.
(4) *Major axis length*: is the length (in pixels) of the major axis of the ellipse that has the same normalized second central moments as the region.
(5) *Eccentricity*: specifies the eccentricity of the ellipse, *i.e.*, the ratio between the minor axis of the ellipse and its major axis.
(6) *Area*: is the actual number of pixels in the region.

Therefore, each region is characterised with thirteen attributes, *i.e.*, Ω_1_ ≡ {*φ_1_, φ_2_, φ_3_, φ_4_, φ_5_, φ_6_, φ_7_*} and Ω_2_ ≡ {*d_1_, d_2_, d_3_, d_4_, d_5_, d_6_*}, where *φ_i_* is associated to the *i*th moment and *d_1_*: *perimeter, d_2_*: *diameter, d_3_*: *minor axis length, d_4_*: *major axis length, d_5_*: *eccentricity, d_6_*: *area* and *φ_i_, d_i_* ∈ [0,1]. The six geometric descriptors are computed for each region following the Equation (2) where *y_i_* represents one of the six geometric descriptors previously defined:

(2) $$d_{i}=\frac{y_{i}-\min\left(y\right)}{\max\left(y\right)-\min\left(y\right)}$$

### 2.3. Classification of Weeds Types

Once each region is characterised by thirteen shape descriptors, the classification stage must decide the class to which each region belongs. For classification, four decision-making methods (CFI, SFI, DES and FMCDM) which have been reported to give excellent results as classifier combiner [46,47], were adapted to combine shape descriptors as attributes. With this aim, they were trained and their different performances were tested to classify monocot *vs.* dicot weeds. The following subsections explain briefly the decision-making methods analysed. Each of the following methods requires a prior step of training in which the method is set to a specific problem (in this case, the classification between monocots and dicots) using a set of positive and negative training examples.

#### 2.3.1. CFI Method

The CFI method requires the computation of the relevance for each attribute, from which the so-called *fuzzy densities* can be computed. The relevance for each attribute is determined by computing the λ − *fuzzy* measure [46]. In the proposed approach, the calculation starts by selecting a set of thirteen fuzzy measures, which will be called *g^1^, g^2^*, …, *g^13^* according to [46]. Each measure represents the individual relevance (strength or competence) of the associated attribute in Ω = Ω_1_∪Ω_2_. The value of λ needed to calculate *g^i^* is obtained as the unique real root greater than −1 of the following polynomial:

(3) $$\lambda +1=\prod_{i\in \Omega }\left(1+\lambda g^{i}\right),\;\lambda \neq 0$$

In the approach proposed for the CFI, the method computes the relevance of each attribute for determining its specific contribution to the decision through the fuzzy densities. The relevance of each attribute is assessed by considering a number of reliable true and false training examples obtained from a set of different regions. The process is as follows: for each region in an image, the grade of support is computed for its class (monocot or dicot), but considering each of the thirteen attributes separately. Thus, the averaged percentage of error, *p_1_*, …, *p_13_*, is obtained for the selected regions and for each attribute, based on the expert criterion. Therefore, the relevance for an attribute *i* is computed by Equation (4):

(4) $$g^{i}=p_{i}/\sum_{j=1}^{13}p_{j}$$

Once the *g^1^*, …, *g^13^* are obtained and λ is found, the CFI is performed following the process described in [48] as follows:

1. For a given region, the vector [*a_1_, a_2_, a_3_, a_4_, a_5_, a_6_, a_7_, a_8_, a_9_, a_10_, a_11_, a_12_, a_13_*]^T^ is obtained; *a_i_* ∈ Ω without loss of generality, assume that *a_1_* is the highest value and *a_13_* the lowest. In this way, this vector is arranged under this criterion, *i.e.*, ∀*i, j, i* > *j, a_i_* > *a_j_* where *i, j* ∈ [1,13].
2. Arrange the fuzzy densities corresponding with the mentioned arrangement, *i.e., g^1^*, …, *g^13^*, and set the first fuzzy density *g*(*1*) = *g^1^*.
3. For *t* = 2 to 13, *g*(*t*) is calculated recursively by Equation (5):
  (5) $$g(t)=g^{i}+g(t-1)+\lambda g^{i}g(t-1)$$
4. Calculate for each candidate region *i*, the final degree of support to be matched with each class *l* as:
  (6) $$S_{i}\left(l\right)=a_{1}+\sum_{h=2}^{13}\left[a_{h-1}-a_{h}\right]g\left(h-1\right)$$
5. The class to which a region belongs is chosen by selecting the maximum support *S_i_(l)* among all classes, in this case two classes, monocots and dicots.

#### 2.3.2. SFI Method

The SFI method is very similar to CFI, coinciding exactly for the first three steps and differing in the way in which the support is estimated, which is reformulated as Equation (7):

(7) $$S_{i}\left(l\right)=\underset{\text{h}\in \left[1,13\right]}{\max}\left\{\min\left\{a_{h},g\left(h\right)\right\}\right\}$$

The decision about the best match is made by selecting the maximum support *S_i_*(*l*) among all classes, in this case monocots and dicots.

#### 2.3.3. DES Theory

The Dempster-Shafer theory (DES) owes its name to works by the both authors in [55,56]. The DES method is applied in our approach following the process described in [46,49] as follows:

(1) A region *l* is matched correctly or incorrectly with its class of weed. Hence, two classes are identified, which are the class of true matches and the class of false matches, *C*_1_ and *C*_2_, respectively. Given a set of samples from both classes, a 13-dimensional mean vector is built, ***v**_i_*, where its components are the mean values of their thirteen descriptors; ***v**_1_* and ***v**_2_* are the mean for *C*_1_ and *C*_2,_ respectively. This process is carried out during a previous phase, equivalent to the training phase in the classification problems.
(2) Given a region *i* and Ω*_i_*, the 13-dimensional vector ***x****_i_* is computed, where its components are the thirteen shape descriptors, *i.e.*, ***x****_i_* = [*e_i1_, e_i2_*, …, *e_i13_*]^T^, *e_ij_* ∈ Ω. Then, the proximity Φ between each component in ***x****_i_* and each component in ***v**_j_* is calculated based on the Euclidean norm ‖·‖ using Equation (8):
  (8) $$\Phi_{jA}\left({\text{x}}_{i}\right)=\frac{{\left(1+{\left\|e_{iA}-{\bar{v}}_{jA}\right\|}^{2}\right)}^{-1}}{\sum_{k=1}^{2}{\left(1+{\left\|e_{iA}-{\bar{v}}_{kA}\right\|}^{2}\right)}^{-1}}\;\text{where}\;A\in \Omega$$
(3) For every class *w_j_* and every region *i*, the membership degrees are calculated according to:
  (9) $$b_{j}^{i}\left(A\right)=\frac{\Phi_{jA}\left({\text{x}}_{i}\right)\prod_{k\neq j}\left(1-\Phi_{kA}\left({\text{x}}_{i}\right)\right)}{1-\Phi_{jA}\left({\text{x}}_{i}\right)\left[1-\prod_{k\neq j}\left(1-\Phi_{kA}\left({\text{x}}_{i}\right)\right)\right]}\;;\;j=1,2$$
(4) The final degree of support that each region *i*, represented by ***x****_i_*, receives for each class *w_j_* is given by:
  (10) $$\mu_{j}\left({\text{x}}_{i}\right)=\prod_{A\in \Omega }b_{j}^{i}\left(A\right)$$
(5) The class to which a region belongs is chosen based on the maximum support received for the class of true matches (*w*_1_), *i.e., max_i_*{μ_1_(***x****_i_*)}.

#### 2.3.4. FMCDM Method

The decision based on the FMCDM method first requires the definition of two elements [57]: (*a*) a triangular fuzzy number *u* as a triplet (*u*_1_, *u*_2_, *u*_3_) and (*b*) a distance (*d*) between two triangular fuzzy numbers *u* and *z*, which can be estimated with Equation (11):

(11) $$d\left(u,z\right)={\left\{\left[{\left(u_{1}-z_{1}\right)}^{2}+{\left(u_{2}-z_{2}\right)}^{2}+{\left(u_{3}-z_{3}\right)}^{2}\right]/3\right\}}^{1/2}$$

Taking into account the thirteen shape descriptors obtained for each region, it can be separated in thirteen groups *C*_1_, *C*_2_, …, *C*_13_. Each group defines a criterion ranging from 0 to 1, *i.e.*, in this approach, there are thirteen criteria available for making the decisions. Assuming there are *m* classes, the fuzzy MCDM paradigm [57,58] can be formulated as the choice of the best alternative *A_i_* (*i* = 1, …, *m*; *m* = 2), where each alternative represents a class. In other words, the FMCDM problem can be expressed in the matrix format as follows:

(12) $$M={\left[x_{ij}\right]}_{m\times n};\;W={\left[w_{j}\right]}_{1\times n};\;i=1,\ldots ,m;j=1,\ldots n$$

*M* is the decision matrix where *x_ij_* is the rating of alternative *A_i_* with respect to the criterion *C_j_*; *w_j_* is the weight assigned to criterion *C_j_*. Shape descriptors are considered as triangular fuzzy numbers so that *x_i_* = (*a_i1_, a_i2_, a_i3_*) ∈ [0,1] where *a_i1_* = *ϕ_i_* − *ε_1_, a_i2_* = *ϕ_i_, a_i3_* = *ϕ_i_* + *ε_2_* and *ε_1_* ≠ *ε_2_*. The random numbers *ε_1_* and *ε_2_* must be no longer than a threshold *T*, which is set to 0.1, one random number must be smaller than the other, and they must not exceed the range [0,1]. The weights associated with each criterion are *w*_1_, *w*_2_, …, *w*_13_, respectively, and are calculated according to the following:

(13) $$w_{h}=\frac{p_{h}}{\sum_{k}p_{k}},\;h,k=1,\ldots ,n$$

where *p_1_*, …, *p_13_* are the averaged percentages of error for each attribute, as in CFI and SFI. Without loss of generality, the values in *x_i_* are ordered so that *a_i1_* ≤ *a_i2_* ≤ *a_i3_*. Therefore, the normalised fuzzy decision matrix (*N*) is obtained as follows:

(14) $$N={\left[r_{ij}\right]}_{m\times n};\;r_{ij}=\left(\frac{a_{i1}}{a_{i3}^{*}},\frac{a_{i2}}{a_{i3}^{*}},\frac{a_{i3}}{a_{i3}^{*}}\right)$$

where a_i3_* = *max_i_*{*a_i3_*}. This equation preserves the property that the ranges of the normalised triangular fuzzy numbers belong to the interval [0,1]. Considering the importance assigned to each criterion, the weighted normalised fuzzy decision matrix (*WN*) is constructed by:

(15) $$WN={\left[v_{ij}\right]}_{m\times n}\;\text{where}\;v_{ij}=r_{ij}w_{j}$$

From *WN*, the elements *v_ij_*, ∀*ij* are normalised positive triangular fuzzy numbers ranging in the closed interval [0,1]. Then, the fuzzy positive-ideal solution *p*^+^ = (1,1,1) and the fuzzy negative-ideal solution *p*^−^ = (0,0,0) are defined. The distances for each alternative can be calculated as follows:

(16) $$d_{i}^{+}=\sum_{j=1}^{n}d\left(v_{ij},p^{+}\right)\;\text{and}\;d_{i}^{-}=\sum_{j=1}^{n}d\left(v_{ij},p^{-}\right)$$

where *d*(·,·) is the distance measured between two fuzzy numbers, defined in Equation (11). According to [57], a closeness coefficient (*CC_i_*) is defined to determine the ranking order of all alternatives, once both *d_i_*^+^ and *d_i_*^−^ for each alternative have been computed. This coefficient is:

(17) $$CC_{i}=\frac{d_{i}^{-}}{\left(d_{i}^{+}+d_{i}^{-}\right)}$$

Obviously, an alternative *A_i_* is closer to the fuzzy ideal solution and farther from the fuzzy negative solution as *CC_i_* approaches +1. Thus, given a region *l*, class *i* is that with the maximum *CC_i_*.

### 2.4. Acquisition System and Images

**Figure 6.**
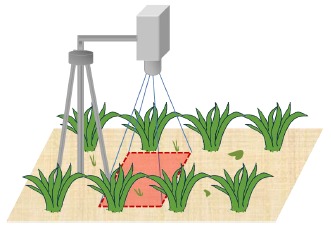
Acquisition process: camera on a tripod at approximately 1.5 m height pointing vertically downward.

The sixty-six images used in this work were taken in maize crops sited in Madrid (Spain) on different days and therefore under varying lighting conditions. A D70 (Nikon, Tokyo, Japan) camera equipped with an 18–70 mm AF-S DX Nikon lens was used to capture images. Image collection was performed by placing the camera on a tripod at approximately 1.5 m height pointing vertically downward (Figure 6).

The images with a dimension of 1700 × 1696 pixels were acquired on the inter-row area, each image covering 0.25 m^2^ (0.5 m × 0.5 m), with a resolution of 72 × 72 dpi. The images were taken with natural lighting. Table 1 summarises the main characteristics of the camera sensor, the images taken and the setting of the camera in the course of the acquisition process.

The multi-zone metering was selected, where the camera sets the exposure automatically to suit the scene by dividing the frame into zones and taking separate readings from each zone. The camera then guesses what parts of the scene are important and chooses the exposure accordingly. This procedure is considered good for landscapes. Furthermore, the camera was left to automatically control contrast, saturation, sharpening and white balance as conditions changed.

## 3. Results

**Figure 7.**
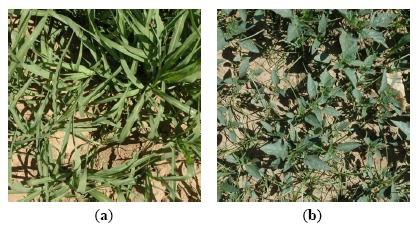
Images showing high levels of infestation of (**a**) monocot weeds and (**b**) a mixture of monocots and dicots.

Regarding the scenes shown in the images used in this work, the vegetation always coincided with weeds (*i.e.*, monocots, dicots or a mixture of both) because the images were taken in the inter-row area. From the sixty-six images available, twenty-eight presented a mixture of weeds, nineteen presented only monocots and nineteen only dicots. A high level of infestation was observed in 14% of the images (Figure 7).

In this work, fifty-six images were selected to represent a wide range of situations. After applying Step 1 (vegetation cover segmentation) and Step 2 (labelling of disconnected regions) in the selected set of images, as described in Sections 2.1 and 2.2, respectively, four hundred different regions were extracted and manually analysed. In general, the number of regions extracted per image ranged from five to twenty. In the cases where an important infestation was observed, fewer than five different regions could be extracted.

**Figure 8.**
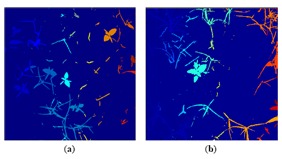
Labelling regions. Each isolated region is identified by a unique colour.

**Figure 9.**
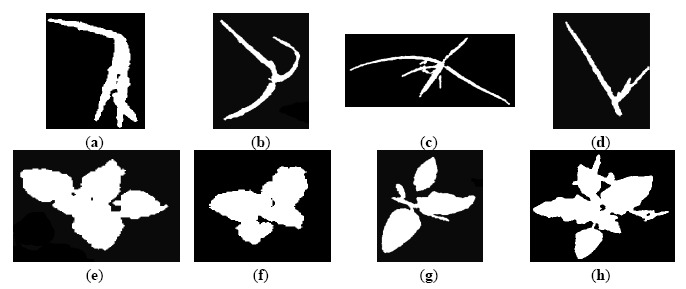
(**a**–**d**) Four regions belonging to monocots; (**e**–**h**) Four regions belonging to dicots.

Figure 8 displays, as an example, the regions extracted by the approach in Steps 1 and 2; concretely, the original image in Figure 4a is represented in Figure 8a. Each region appears labelled with a unique label, represented by a colour in a scale for visualisation purposes. Figure 9 represents different regions belonging to weed classes where the regions' structure plays a key role in the proposed discrimination process.

The tests corresponding to the proposed decision-making strategies (CFI, SFI, DES and FMCDM) were carried out with fifty-six images including four hundred different regions belonging to monocots and dicots. Sixteen of the images containing one-hundred and fourteen regions were used for computing the relevance of each attribute for CFI and SFI based on Equation (4), the mean vectors for DES, as explained in Section 2.3.3, and the weights for FMCDM, as described in Equation (13).

At this point, the information of class membership provided by the expert criterion was available. Thus, the correct class for each region in an image was known according to the expert knowledge, and this information was used to compute the percentage of error of the proposed approach. The averaged percentages of error, *p_1_*, …, *p_13_*, were *p_1_* = 18 (*ϕ_1_*), *p_2_* = 20 (*ϕ_2_*), *p_3_* = 30 (*ϕ_3_*), *p_4_* = 28 (*ϕ_4_*), *p_5_* = 24 (*ϕ_5_*), *p_6_* = 23 (*ϕ_6_*), *p_7_* = 21 (*ϕ_7_*), *p_8_* = 27.5 (*d*_1_), *p_9_* = 27.5 (*d*_2_), *p_10_* = 27.5 (*d*_3_), *p_11_* = 15 (*d*_4_), *p_12_* = 40 (*d*_5_) and *p_13_* = 40 (*d*_6_). Based on Equation (4), the fuzzy values g*^1^*, …, g*^13^* were obtained. From Equations (4) and (13), *w_i_* = g*^i^, i* = 1, …, *n*. Finally, considering the true and false matches under the expert knowledge, the mean vectors ***v**_1_* and ***v**_2_*, were obtained and normalized from 0 to 1.

The best individual results, according to the thirteen shape descriptors, were obtained with the first, second and seventh Hu moments and the geometric shape descriptor named major axis length. The worst attributes in terms of percentage were eccentricity and area, respectively. These attributes do not contribute in any way (positive or negative) to the final decision.

At a second stage, for each of the two hundred eighty-six regions obtained from the remaining forty images used for testing, the proposed decision-making strategies, CFI, SFI, DES and FMCDM as described in Sections 2.3.1, 2.3.2, 2.3.3 and 2.3.4, respectively, were applied, and the success for each region, as well as the average of these hits, were computed.

Based on the best individual results, the four stages-approach proposed was applied again with only these four shape descriptors (*ϕ_1_, ϕ_2_, ϕ_7_* and *d_4_*—major axis length). This decision was accomplished because some attributes do not contribute to the final decision. The proposed decision-making strategies were applied in the same way as described in Section 2.3, but in this case taking into account four shape descriptors. The results show that the strategies based on combining the best attributes, improve the accuracy six points average. The reason is none other than some shape descriptors do not characterise properly the regions belonging to the two weeds species aim of study. For this reason they do not contribute to take the right decision.

Table 2 displays the averaged classification accuracy and standard deviations obtained with the four decision-making strategies when the seven Hu moments and six geometric shape descriptors take part in the final decision (see first column). Based on the individual results explained above, second column shows the results obtained with the four decision-making strategies when only take part in the final decision the best shape descriptors (*ϕ_1_, ϕ_2_, ϕ_7_* and *d_4_*). Finally, for comparative purposes SVM was tested as described in [51,52]. The Gaussian Radial Basis Function (RBF) kernel was used for both training and testing phases. RBF outperforms the other two kernels tested: Polynomial and Sigmoid [51]. These results are also presented in Table 2.

The combined strategy showed the best results for FMCDM. Similar results were observed for CFI and SFI in terms of percentage and low standard deviation. Although SVM has good generalization performance and a fast decision computation once trained, it has some drawbacks, e.g., the selection of the kernel function parameters, the problem of over-fitting from optimising the parameters to model selection and the robustness of the SVM-based approach against illumination variability. Taking into account the final requirements of our problem as described in Section 1, the results are considered good enough to solve the decision problem. The strategy proposed has been implemented in Matlab. The processing time with the best proposal is less than 0.5 s, which is enough to achieve real-time processing even though Matlab is not generally used to do real-time analysis. The image acquisition frequency is around 6 fps and is sufficient because our proposal guarantees overlapping between images, considering the treatment speed (around 1.6 m/s).

## 4. Conclusions

This paper proposes a strategy for discriminating between monocot and dicot weeds. The method, which has proven effective and simple, is based on colour segmentation, morphological operations and well-known shape descriptors and classifiers, common operations in image processing.

The shape descriptors and the classifier are the two most important factors affecting the performance of the proposed approach. The Hu moments have been shown to be relevant in shape recognition processes because they are invariant to translation, rotation and scaling. Accordingly, for each region in an image the seven Hu moments are obtained to characterise the region in order to discriminate between monocot and dicot weeds. With the same aim, six geometric shape descriptors (*perimeter, diameter, minor axis length, major axis length, eccentricity* and *area*) were proposed. Under the FMCDM method, the values of the best descriptors are combined and a decision for choosing the unique class for each region is made. This strategy outperforms the SVM, CFI, SFI and DES combined decision-making methods.

The proposed combined strategy works properly when the weeds present an early stage of growth, which coincides with the right timing for herbicide application. If the crop is further developed, the weeds will most likely present overlapping and the segmentation process will become difficult, mainly due to occlusions leading to incorrect differentiation of the weed shapes. Nevertheless, the proposed approach provides a useful methodology to discriminate seedlings of monocots and dicots in real field situations, consequently, which display a mixture of both types.

Although the results can be considered satisfactory, better results could be obtained by improving the segmentation stage where a greenness index was calculated to distinguish vegetation against non-vegetation, and the selection of descriptors able to characterise successfully regions in order to discriminate between weeds species. The four decision making strategies proposed cope with the variability of lighting conditions such as it was observed in previous works [48–50]. However, an improvement in the accuracy is desirable. Other classifiers or combining classifiers may be applied to automate the classification [46,47], using induced knowledge based on shape descriptors.

As future work, it is proposed to improve the processing time of the system. Taking advantage of the overlapping of the images it would be possible to get better results during the segmentation stage. As it was mentioned in Section 1, the final aim is to integrate a RGB camera as part of the sensors systems on board an autonomous tractor in order to reach the weed classification in real time.

The proposed combined approach can be extrapolated to any situation where monocots and dicots are present, e.g., to discriminate between maize (a monocot crop) and dicot weeds. Moreover, it can be used in images obtained by low-altitude UAVs. In this context, site-specific weed management could significantly reduce herbicide use, with undoubted benefits for the environment. In addition, efficiency is higher if selective treatment is performed for each type of infestation instead of using a broadcast herbicide. In summary, this proposal improves a utility that is essential for the future of the site-specific weed management.

## Figures and Tables

**Table 1.** Several important characteristics of the camera sensor, the images taken and the setting of the camera used in the acquisition process.

**Element**	**Property**	**Value**
Sensor	Sensor type	CCD RGB—23.7 mm × 15.6 mm
	Total pixels	6.24 million
	Crop factor (35 mm)	≅1.5

Image	Bit depth	24
	Resolution unit	2
	Colour representation	sRGB
	Compressed bits/pixel	4

Camera	Relative aperture	f/9
	Exposure time	1/320 s
	Focal length	29 mm
	Max. aperture	3.9
	Metering mode	Multi-zone

**Table 2.** Averaged classification accuracy and standard deviations obtained for the SVM, CFI, SFI, DES and FMCDM decision-making strategies.

**Decision Based on # Shape Descriptors**	**All of Them (13)**		**The Best Ones (4)**	

**Decision Making Strategies**	**%**	**σ**	**%**	**σ**
SVM	82.8	2.1	87.1	2.0
CFI	84.2	1.7	90.2	1.7
SFI	84.1	1.5	90.1	1.6
DES	81.9	2.3	89.1	2.2
FMCDM	85.8	1.8	92.9	1.7
